# Zika Virus: a Review from the Virus Basics to Proposed Management Strategies

**DOI:** 10.4084/MJHID.2016.056

**Published:** 2016-11-01

**Authors:** Lorenzo Zammarchi, Michele Spinicci, Alessandro Bartoloni

**Affiliations:** 1 Dipartimento di Medicina Sperimentale e Clinica, Università di Firenze, Florence, Italy; 2 SOD Malattie Infettive e Tropicali, Azienda Ospedaliero Universitaria Careggi, Florence, Italy

## Abstract

This review aims to summarize the body of knowledge available on Zika virus to date. A comprehensive review of the scientific literature on Zika virus was performed with the aim to stress relevant aspects for healthcare professionals in the non-endemic areas. For several years, the Zika virus infection was considered an extremely rare exotic disease with poor clinical relevance. However, Zika virus has recently gained the attention of the scientific community and public opinion since the virus spread to the Pacific islands and the South America in an unprecedented epidemic, and additionally due to the definitive evidence that the infection could be complicated by Guillain-Barré syndrome, passed through vertical transmission, and result in central nervous system abnormalities (including microcephaly) of the fetus. Studies and scientific evidence on the complications associated with Zika virus infection are growing day by day. It is advisable that the healthcare professionals working in non-endemic areas maintain full awareness on this issue in order to practice proper management of the imported cases of Zika virus infection.

## Introduction

For about 60 years after its first isolation, which occurred in 1947 from a rhesus monkey in the Zika Forest, Uganda,^1^ the Zika virus (ZIKV) infection has been considered responsible for an extremely rare exotic disease of poor clinical relevance and limited public health importance. However, since the virus spread to the Pacific islands and the South America, causing an unprecedented epidemic, it has received the attention of the scientific community along with public opinion, due to the definitive evidence that the infection is associated with Guillain-Barré syndrome (GBS) and, when contracted during pregnancy, with microcephaly and other abnormalities of the central nervous system of the fetus.^2^

## Etiologic Agent

ZIKV is an arbovirus (arthropod borne viruses), and it is transmitted by mosquitoes of the genus *Aedes*. It belongs to the family Flaviviridae, genus *Flavivirus*, with little genomic divergence from other viruses of the same genus.^3^ The genome of ZIKV consists of a single-stranded positive sense RNA molecule of 10794 kb in length, and a single central open reading frame encoding for a polyprotein, between two non-coding regions.^4^ The polyprotein is cleaved into a capsid protein, a precursor of the membrane protein, a protein for the envelope, and seven non-structural proteins.^4^ Based on the sequencing of the entire genome, two main lineages were identified, i.e. the African lineage, further divided into two groups (clusters Uganda and Nigeria) and the Asian lineage, recently also called Asian/American.^3^ Most of the strains belonging to the African lineage were isolated from enzootic vectors, whereas the Asian lineage has been associated with the major human epidemics reported until now.^3^ The strains recently isolated in the Americas (Brazil, Puerto Rico, Haiti, Guatemala, Suriname) show a rapid and wide genetic divergence, probably linked to the spread of the virus into an immunologically naïve population.^3^ Some authors suggest that the high rate of complications of ZIKV infection not described previously (namely microcephaly and GBS), could be due to the phenotypic variations of new strains that have originated from the Asian lineage. For example, the new strains could induce a higher viremia, fostering the transplacental transmission and the generation of the human reservoir, or they could manifest a greater neurotropism.^3^

## Life Cycle, Reservoirs and Transmission Route

ZIKV infection is mainly transmitted through a vectorial route by the bite of mosquitoes belonging to the genus *Aedes*.^3^ Before it spread to the Pacific Islands and the Americas, ZIKV was maintained in nature through a sylvatic cycle, which is probably still the case in Africa.^5^ In Africa, enzootic transmission is prevalent; non-human primates are the main reservoirs (maybe with other vertebrates such as small mammals, birds, and reptiles) and the vectors are various species of mosquitoes that proliferate preferably in forests, such as *Ae. furcifer*, *Ae. luteocephalus*, *Ae. africanus*, *Ae. vittatus*, *Ae. taylori*, *Ae. dalzieli*, *Ae. hirsutus*, *Ae. metallicus*, *Ae. unilinaetus*, *Mansonia uniformis*, *Anopheles coustani* and *Culex perfuscus*.^5^,^6^ Human cases in this setting are sporadic. Conversely, the urban cycle has sustained the epidemics reported in Micronesia in 2007, in French Polynesia in 2013–2014, as well as the current outbreak in the Americas.^7^–^9^ In these cases, humans are the main reservoir, whereas the vectors are different mosquitoes of the genus *Aedes,* such as *Ae. hensilli, Ae. polynesiensis* and *Ae. aegypti* that have had (or still have) a role in the epidemics in Micronesia,^10^ French Polynesia^11^ and the Americas,^6^ respectively. Furthermore, there is laboratory evidence that *Ae. albopictus* (including the Italian strain) can be a competent vector for ZIKV,^12^,^13^ though less effective than *Ae. aegypti*.^13^,^14^ To date, *Ae. albopictus* has been only suspected to have played a role in the epidemic occurred in Gabon in 2007 when the virus was detected both in the serum of febrile individuals and in mosquitoes of this species.^15^ In that case, the ZIKV strain belonged to the African lineage.^15^

A growing body of evidence indicates that the virus can be transmitted during pregnancy through the placenta and within the perinatal period.^16^,^17^ Indeed, viral RNA was detected by the Polymerase Chain Reaction (PCR) in the amniotic fluid, the fetal tissues, and the central nervous system of newborns from women who contracted the infection in pregnancy.^17^,^18^ There are a number of case reports demonstrating the possibility of sexual transmission from an infected male to his partners (both female and male), both in the case of the symptomatic and asymptomatic disease.^19^–^21^ The virus was detected in the sperm up to 24 days after infection by the viral isolation technique and after 188 days by PCR.^22^ Recently, the first case of female-to-male sexual transmission of ZIKV was reported in the United States,^23^ and ZIKV RNA has been detected in vaginal fluids 3 days after symptom onset, and in one case, in cervical mucus up to 11 days after symptom onset.^24^ Moreover, the virus can potentially be transmitted through blood transfusions and other substances of human origin (SoHO).^25^ There are also a few cases due to laboratory accidents,^26^ and a single report of suspected transmission due to a monkey bite.^27^ Aside from serum, the virus has also been isolated from urine, saliva, breast milk and semen; although the possibility of transmission through saliva, breast milk, and urine has not been demonstrated to date.^28^–^31^

## Epidemiology

ZIKV was detected for the first time in the blood of a rhesus monkey in 1947 at the Yellow Fever Research Institute in Entebbe, Uganda, and again in the following year, in the vector *Ae. africanus*.^1^ The diagnosis of the first human case dates back to 1954, in Nigeria, during the investigation of a jaundice outbreak due to Yellow fever.^32^ After its discovery, the circulation among humans of ZIKV in various areas of Africa and Asia was confirmed by a few case reports and some seroprevalence studies.^7^,^33^ The first large outbreak was described in 2007, in the Federal State of Yap islands in Micronesia, where ZIKV infected about 5,000 people, corresponding to 75% of the population.^34^ In Thailand, East Malaysia, Cambodia, the Philippines and Indonesia, sporadic cases were reported in the following years.^33^ Between October 2013 and April 2014, a second large outbreak affected French Polynesia, where it is estimated that 32,000 people were infected (11% of the population).^7^ Subsequently, the virus spread in many other Pacific Islands such as the Cook Islands, Easter Island and New Caledonia, the Solomon Islands and Vanuatu.^33^ At the beginning of 2015, ZIKV reached the Americas for the first time, where it was first reported in Brazil’s northeastern State of Rio Grande do Norte,^9^ and in a traveller returning to Italy who visited Bahia in the same period.^35^ Briefly, the virus has spread throughout most of the Brazilian States, causing, as of February 2016, between 500,000 and 1,500,000 infections, according to data from the Brazilian Ministry of Health.^36^ Since October 2015, Colombia also began to report indigenous cases, followed by many other states of the Americas.^37^ Currently the World Health Organization indicates the presence of a continuous vectorial transmission in 60 countries and territories, including most of the states of Central and South America, many countries in South East Asia, Oceania and the Pacific and, with regard to Africa, Cape Verde and more recently, Guinea Bissau.^2^,^38^ In July 2016, several autochthonous cases were reported in Florida, United States.^39^ The list of countries with current autochthonous transmission of ZIKV is available on the website of the European Centre for Disease Prevention and Control: http://ecdc.europa.eu/en/healthtopics/zika_virus_infection/zika-outbreak/Pages/Zika-countries-with-transmission.aspx.

The risk to be infected while travelling seems quite small. According to data from the CDC, in USA among 3,335 pregnant women who moved to- or lived in areas at risk and were tested for ZIKV, only 0.8% tested positive; a positive result occurring in 2.9% among those who had at least a typical symptom of ZIKV, and 0.3% of those which remained asymptomatic.^40^

## Clinical Manifestations and Pathogenesis of Complications

The infection is asymptomatic in about 80% of cases.^8^ The incubation period in symptomatic cases is not precisely known. According to a systematic review, the median incubation period is 5.9 days and 95% of individuals develops symptoms 11.2 days after the exposure.^41^ According to the European Centre for Disease Control and Prevention (ECDC) case definition, exposure in an area with transmission of ZIKV within two weeks prior to the onset of symptoms is considered epidemiological criteria for diagnosis, as the incubation period is likely less than 14 days.^42^ The infection acquired vectorially or by sexual contact has, in almost all cases, a benign course without complications leading to resolution of symptoms within a period of about two weeks. The most common manifestation (90% of cases) is a rash, typically macular or maculopapular, often itchy and with centrifugal evolution (from the trunk to the extremities), that generally lasts 4–5 days (range: 2–14 days).^7^,^8^,^33^ In about 65–70% of cases, the rash may be preceded by 1–4 days of fatigue and fever (generally lower than 38 degrees) for maximum 7 days.^7^,^43^ The third most frequent symptom is arthralgia (65% of cases), eventually associated in 20–45% of cases with periarticular edema (hands and feet, and less frequently knees and wrists), which generally persist for a week (up to a month).^7^ Bilateral, not purulent conjunctival injection may occur in approximately 55–60% of cases, and it typically resolves in 1–2 weeks.^7^,^8^ Other manifestations include headache, myalgia, and retro-orbital ocular pain. In a case series of Brazilian pregnant women with ZIKV infection, localized or generalized lymphadenopathy was found to be a relatively common clinical manifestation (40% of cases), whereas it was reported in only 15% of cases during the French Polynesia outbreak.^7^

Some authors speculate that the high phenotypic variation rate of the strains belonging to the Asian lineage isolated in the Americas could result in infections characterized by a greater neurotropism or a higher viremia, thus fostering the transplacental transmission.^3^ Two different mechanisms were hypothesized to explain the pathogenesis of neurological complications and the congenital infection: a direct viral damage and an immune-mediated mechanism.^44^ Most of the studies conducted so far have investigated the former. Studies in animal models (rat) showed that ZIKV is extremely neurotropic 45 and that it could be transmitted through the placenta, inducing growth retardation, fetal death, placental damage, apoptosis of neural progenitor cells, and impaired neural proliferation and development in affected fetus.^46^–^48^ Concerning the immune-mediated hypothesis, only slight differences in cytokine levels between acute and convalescent samples have been observed in a small cohort of patients.^49^ Another interesting *in vitro* observation is that plasma immune to virus (DENV) was able to enhance ZIKV infection probably through a mechanism of antibody dependent enhancement (ADE).^50^ According to the ADE mechanism, which has been hypothesized for DENV, antibodies generated during a primary infection with DENV will not be of sufficient concentration or avidity to neutralize a secondary infection with DENV of a different serotype, however, they might still opsonize the secondary virus and thus drive higher viral loads.^50^ This observation suggests that subjects with previous DENV immunity could have more severe ZIKV clinical manifestations or increased risk of transplacental transmission compared with subjects without previous DENV infection.

The correlation between ZIKV infection and GBS was observed for the first time in 2013–2014 during the ZIKV outbreak in French Polynesia. The outbreak registered 42 cases in 4 months, compared to an average of 5 cases per year before 2013.^29^ A study, reporting data from French Polynesia epidemic, showed that the risk of developing GBS after ZIKV infection is around 2.4 per 10,000 infections Zika virus, similar to the risk following a *Campylobacter jejuni* infection.^43^ The appearance of GBS occurred after a period ranging from 2 to 23 days (median 6 days) after the onset of the symptoms of infection.^7^ The typical electromyography pattern found in these patients was characterized by acute motor axonal neuropathy, without the ganglioside antibodies that are characteristic of this condition.^51^ At present, in addition to French Polynesia, 12 other countries affected by a ZIKV epidemic reported at least one case of GBS in patients with recent infection, and 8 reported a significant increase compared to previous years.^52^ In particular, in Brazil from January to November 2015 1,708 cases were registered, corresponding to an increase which varied among the states ranging from +526.7% (Alagoas) to +60.9% (Rio de Janeiro).^53^

The correlation between ZIKV and microcephaly and other damages due to congenital infection was first observed in Brazil. In October 2015, about seven months after the identification of the first cases of ZIKV in the northeast of Brazil, the Ministry of Health of Brazil reported an unusual increase in cases of microcephaly in the states of Pernambuco, Paraiba and Rio Grande do Norte, with an evident geographical overlap with the epidemic of ZIKV.^54^

In November 2015, in the state of Paraiba, ZIKV RNA was detected in the amniotic fluid of two women whose fetus had an ultrasound diagnosis of microcephaly.^55^ These findings induced the Brazilian Ministry of Health, the Centers for Disease Control (CDC), and the ECDC to issue specific alerts on the possible association of microcephaly and ZIKV infection.^56^,^57^ On February 1, 2016, following the rise in cases of microcephaly and GBS in the areas of ZIKV spread, the WHO declared ZIKV a Public Health Emergency of International Concern.^58^ An increase of 20-times in the frequency of microcephaly (20 cases per 10,000 live births) was found by comparing the historical statistics of this disorder in Brazil with those of 2015.^59^ On the other hand, current incidence of microcephaly in Brazil could be partially overestimated due to a previous under-diagnosis bias and a new surveillance protocol, which may have a higher sensitivity in detecting cases.^59^,^60^ Between October 2015 and June 30, 2016, Brazil reported 8,165 suspected cases of microcephaly and other nervous system disorders suggestive of congenital infection.^38^ Of these, 1,638 are confirmed cases of microcephaly, 270 of which have been laboratory-confirmed for ZIKV infection.^38^ An increase in cases of microcephaly has been found retrospectively also in French Polynesia after the epidemic of 2013–2014.^61^ A retrospective study, based on a mathematical model and on data collected in French Polynesia, concluded that the risk of microcephaly was 1% for newborns of women who contracted the infection during the pregnancy, and the most dangerous period was the first trimester of gestation.^61^ According to a second mathematical model built on Brazilian data, microcephaly could complicate from 0.88%–13.2% of pregnancies when the woman is infected in the first trimester.^62^

An observational study in Brazil reported fetal abnormalities in 29% (12 of 42) of the pregnant women infected with ZIKV, and in none of the 12 pregnant women who tested negative.^63^ The anomalies included fetal deaths at 36 and 38 weeks of gestation (2 foetuses), intrauterine growth restriction (IUGR) with or without microcephaly (5), ventricular calcifications or other disorders of the central nervous system (7 fetuses), abnormalities of the amniotic fluid volume, or the brain or umbilical artery flow (7 fetuses).^63^

In addition to Brazil and French Polynesia, several other countries reported autochthonous (Colombia, Cape Verde, El Salvador, French Guiana, Marshall Islands, Martinique, Panama, Puerto Rico) or imported cases (USA, Spain, Slovenia, all in women who had travelled in Central and South America) of malformations of the central nervous system due to ZIKV congenital infection.^38^ Clinical data on infants with congenital infection are severe. A study conducted in the state of Pernambuco analysed 23 babies with microcephaly whose mothers reported symptoms compatible with ZIKV infection in the first or second trimester.^64^ Seven underwent lumbar puncture with detection of IgM anti ZIKV in the cerebrospinal fluid. All the children had significant severe brain abnormalities noted at the CT scan, such as deficiency in brain development, brain calcifications (mainly in the corticomedullar junction), global hypogyration of the cerebral cortex, white matter abnormalities, and significant ventriculomegaly.^64^ Moreover, almost a third of infants with microcephaly related to ZIKV congenital infection had ocular abnormalities, such as pigment accumulation in the retina and chorioretinal atrophy, abnormalities of the optic nerve, bilateral coloboma of the iris, and lens subluxation.^65^

Reports of other severe or life-threatening events are exceptional and almost always limited to patients with extreme age or comorbidities. Among those, there are 4 cases of neuroinvasive disease (a case of meningoencephalitis in an elderly,^66^ a case of myelitis in a child,^67^ two cases of encephalopathy^68^) and two fatal cases in patients with severe comorbidities (a child with sickle cell disease 69 and a man with alcoholism, systemic lupus erythematosus, and rheumatoid arthritis in chronic treatment with corticosteroids^70^).

## Diagnosis

The diagnosis is based on serological and molecular tests, in addition to clinical and epidemiological criteria. The differential diagnosis of ZIKV infection may be challenging since signs and symptoms are nonspecific and mimic other infections and in particular other arbovirosis such as dengue and chikungunya which have similar clinical presentation and epidemiological distribution.^71^,^72^ Moreover the accuracy of the serological tests for ZIKV, including the reference test of neutralization, is burdened by a high degree of cross-reactions with other flaviviruses.^19^,^72^,^73^

Furthermore, individuals with previous infections or immunizations by flavivirus may show the “antigenic original sin” phenomenon, i.e. they can present a specific antibody response to the virus which is responsible for the previous infection rather than to the current one.^74^ A commercial ELISA test that uses the NS1 antigen of ZIKV shows good performance, including in terms of specificity.^75^ IgG seroconversion occurs, on average, 9 days after the onset of symptoms, whereas the IgM may appear 4–5 days after the onset.^41^

With regard to the virological testing, PCR is the most used technique. PCR remains positive in serum in the first few days of symptoms (usually <7 days).^76^ The persistence of viral RNA in saliva is similar, although some studies reported that it can last up to one month.^29^ In urine, the persistence has been demonstrated up to 10–20 days after manifestation.^76^,^77^ A recent study showed that ZIKV RNA may be detected by PCR in whole blood for a longer period than in serum and urine (up to 2 months post-symptom onset).^78^ In one case, a pregnant woman (whose fetus had major malformations and congenital ZIKV infection) was still viremic at both 4 and 10 weeks after the onset of symptoms, suggesting a longer-lasting viremia in pregnant women.^79^

In the presence of a laboratory confirmed diagnosis during pregnancy and / or ultrasonographic evidence of fetal damage suggestive for fetal infection, PCR testing for ZIKV on amniotic fluid can be performed through amniocentesis.^17^ Some experts recommended, similarly to other infections in the TORCH group, waiting until at least 21 weeks of gestation and 6 weeks after the onset of symptoms in the mother before performing amniocentesis.^80^ The case definition of ZIKV according to the ECDC is reported in Table 1.

## Clinical Management of Pregnant Women

Several institutions, like the CDC and ECDC, advise pregnant women against travelling to areas where the transmission of ZIKV is reported.^38^,^81^ If the travel is indispensable, the woman should pay attention to the prevention of insect bites, based products.^82^ Both symptomatic and asymptomatic pregnant women exposed to the risk of infection (those returning from areas where transmission of Zika virus is known to occur or those who have had sex with males returning from areas at risk), should undergo a laboratory test for Zika.^83^ Several flow charts for testing pregnant women have been proposed.^80^,^83^–^85^ In general, the screening test to be used for pregnant should be selected according to the presence or absence of symptoms and the time elapsed since the potential exposure. Given the kinetics of the viro-immunological markers of infection, it is worthwhile to use both serological tests and PCR on serum, saliva, and urine if the tests are performed within one month from the onset of symptoms or since the last exposure.^76^ After more than one month has passed, serology alone may be sufficient, though the long duration of viremia in pregnant women could support the use of PCR on serum even after a longer period has elapsed. It should be noted that the IgM might be negative even in the case of recent infection,^84^ probably due to the “antigenic original sin” phenomenon.^74^,^86^ In these cases, the diagnosis is based on positivity of especially during the day, using icaridin or DEET IgG and PCR.^84^ In the presence of a positive test for ZIKV, the woman should undergo an obstetric ultrasound follow-up every 3–4 weeks.^83^ The option to perform amniocentesis in order to detect viral RNA by PCR,^83^ mentioned previously, should be considered and discussed with the woman, even though data are not present on the sensitivity of this technique or on its prognostic value in case of a positive result (i.e. it’s unknown if positive PCR necessarily lead to fetal and / or neonatal disease). Moreover, the presence of microcephaly cannot be accurately assessed with ultrasounds before the 3rd trimester of pregnancy.^80^ Newborns from ZIKV infected women should undergo strict neonatology and pediatric controls, including serological and molecular testing for ZIKV.^83^,^84^,^87^

A pathological evaluation of fetal tissue specimens (e.g., placenta and umbilical cord) may be used to establish the presence of maternal Zika virus infection and can provide a definitive diagnosis for pregnant women with a ZIKV infection whose serology results were inconclusive due to possible cross reactions with other flaviviruses.^83^

## Therapy and Vaccine

No therapies are available to date. The epidemiological and clinical experience obtained during the current outbreak makes the development of a ZIKV vaccine a global health priority. Promisingly, a recent study showed that a single immunization with a plasmid DNA vaccine or a purified inactivated virus vaccine could provide complete protection against ZIKV challenges in susceptible mice.^88^ However, the achievement of a ZIKV vaccine for humans requires a better insight into ZIKV immunology and mechanisms of immune protection. For example, the development of a live-attenuated vaccine is inadvisable until the link between GBS and ZIKV will be elucidated and additional complications related to pre-existing flavivirus exposure should be deeply probed in order to develop an efficient and safe vaccine.^89^

## Control Strategies in non-Endemic Area

Much of the world’s tropical and sub-tropical regions are at risk for further ZIKV spread. In temperate zones, such as Europe, the emergence of autochthonous cases or small foci is possible anywhere competent vectors, basically, mosquitoes of *Aedes* genus, are present. In Europe, the presence of *Ae. aegypti* is currently limited to Madeira, the eastern coast of the Black Sea, and the Netherlands.^90^ However, *Ae. albopictus* mosquitoes, which are proven competent vector for ZIKV,^13^ albeit less efficient than *Ae. aegypti,* are widespread in several countries around the Mediterranean basin, especially in Italy.^90^ *Ae. albopictus* was implicated in the autochthonous cases of dengue fever reported in 2010 in Croatia,^91^ in 2010, 2013, and 2015 in France,^92^–^94^ and of chikungunya in Italy in 2007,^95^ in France in 2014,^96^ and in Spain in 2015.^97^ In non-endemic areas, the programs for arthropod-borne disease control, including ZIKV, have been based on the experience gained in the last decades in controlling the transmission of dengue and other arboviruses, and they are mainly focused on early detection and reporting of imported cases and vector control measures^98^ in accordance with national strategies. For example, in Italy suspected cases must be notified to the Public Health Authorities within 12 hours during the period of activity of *Ae. albopictus* (from June to October) or within 24 hours during the rest of the year. In the period of vector activity, patients with suspected or confirmed diagnosis who are not hospitalized, should be advised to remain isolated at home.^85^ Public health authorities should immediately activate rapid vector control measures as soon as imported cases are detected, in order to implement pest control methods around the residence of the index case and the other sites that infected patient visited. In France, where sporadic autochthonous cases of arboviruses have recurred periodically in the summer months since 2010, the response includes epidemiological and entomological door-to-door surveys around the index case residence in order to detect further infections by an active case finding, and to guide vector control measures (elimination of possible larval breeding sites, spraying of deltamethrin) in the outbreak areas.^92^

Despite that the main route of ZIKV transmission is via the *Aedes* mosquitoes, strong evidence has been demonstrated that ZIKV can be transmitted by sexual contact, including vaginal, anal and oral sexual intercourse. Based on the available knowledge, the CDC and WHO issued guidance papers on precautions to be kept: people returning from areas where local transmission of Zika virus is known should adopt safer sex practices or consider abstinence for at least 8 weeks upon return, and up to 6 months if the male partner is symptomatic; couples planning a pregnancy should wait for the same duration before attempting to conceive.^99^ Men exposed to risks of infection should avoid unprotected sexual intercourse for the whole length of the gestation if their partner is pregnant.^99^,^100^

Moreover, a potential risk of ZIKV transmission through SoHO has been identified; competent authorities and healthcare professionals need to be vigilant regarding the risk of donor-derived ZIKV transmission. A document issued by the ECDC based on previous experiences, such as for West Nile Virus, is now available to support the implementation of national preparedness plans for the safety of SoHO, with respect to ZIKV infection, in both affected and non-affected area.^101^

## Figures and Tables

**Table 1:** Case definition for surveillance of Zika virus infection according to the European Centre for Disease Control and Prevention. Available at: http://ecdc.europa.eu/en/healthtopics/zika_virus_infection/patient-case-management/Pages/case-definition.aspx?preview=yes&pdf=yes#sthash.WAzTC7zm.dpuf

Classification	Probable case A person meeting the clinical criteria and the epidemiological criteria. A person meeting the laboratory criteria for a probable case Confirmed case A person meeting the laboratory criteria for a confirmed case
Clinical criteria	A person presenting with a rash, with or without fever and at least 1 of the following signs and symptoms: Arthralgia or Myalgia or Non-purulent conjunctivitis/hyperaemia
Laboratory criteria	Laboratory criteria for a probable case: Detection of Zika specific IgM antibodies in serum Laboratory criteria for a confirmed case (at least 1 of the following): Detection of Zika virus nucleic acid in a clinical specimen Detection of Zika virus antigen in a clinical specimen Isolation of Zika virus from a clinical specimen; Detection of Zika virus specific IgM antibodies in serum sample(s) and confirmation by neutralization test Seroconversion or four-fold increase in the titer of Zika specific antibodies in paired serum samples
Epidemiological criteria	History of exposure in an area with transmission of Zika virus within two weeks prior to onset of symptoms or Sexual contact with a male having been confirmed with a Zika virus infection in the past four weeks or Sexual contact with a male who had been in an area with Zika virus transmission in the past four weeks A list of Zika affected areas is kept updated on the ECDC website: http://ecdc.europa.eu/en/healthtopics/zika_virus_infection/zika-outbreak/Pages/Zika-countries-with-transmission.aspx
